# A trial on unruptured intracranial aneurysms (the TEAM trial): results, lessons from a failure and the necessity for clinical care trials

**DOI:** 10.1186/1745-6215-12-64

**Published:** 2011-03-04

**Authors:** Jean Raymond, Tim E Darsaut, Andrew J Molyneux

**Affiliations:** 1 Centre hospitalier de l'Université de Montréal (CHUM), Notre-Dame Hospital, Department of Radiology and Interventional Neuroradiology Research Unit, 1560 Sherbrooke east, Pav. Simard, Z12909, Montreal, Quebec, H2L 4M1, CANADA; 2 Oxford Neurovascular and Neuroradiology Research Unit, Level 6, West Wing, John Radcliffe Hospital, Headley Way, Oxford OX3 9DU, UK

## Abstract

The trial on endovascular management of unruptured intracranial aneurysms (TEAM), a prospective randomized trial comparing coiling and conservative management, initiated in September 2006, was stopped in June 2009 because of poor recruitment (80 patients). Aspects of the trial design that may have contributed to this failure are reviewed in the hope of identifying better ways to successfully complete this special type of pragmatic trial which seeks to test two strategies that are in routine clinical use. Cultural, conceptual and bureaucratic hurdles and difficulties obstruct all trials. These obstacles are however particularly misplaced when the trial aims to identify what a good medical practice should be. A clean separation between research and practice, with diverging ethical and scientific requirements, has been enforced for decades, but it cannot work when care needs to be provided in the presence of pervasive uncertainty. Hence valid and robust scientific methods need to be legitimately re-integrated into clinical practice when reliable knowledge is in want.

A special status should be reserved for what we would call 'clinical care trials', if we are to practice in a transparent and prospective fashion a medicine that leads to demonstrably better patient outcomes.

## Background

Clinical research can be extremely difficult, especially when the aims are to appraise the real value of interventions that are widely judged to be justified by common sense, but that have yet to be proven effective or beneficial. This situation is common; it leaves a lot of room for error and patient harm, on a large scale. Hence few medical interventions need to be studied with more rigour and few deserve more support (from patients, physicians, agencies or institutions), than trials which aim to determine the value of commonly performed prophylactic surgical interventions. This becomes particularly pertinent when the intervention carries a small but definite risk of causing death or disability in asymptomatic individuals.

Yet the current clinical research environment has built a system that makes such studies virtually impossible, as will be exemplified here.

The Trial on Endovascular Aneurysm Management (TEAM) was such an enterprise that failed. We will first review the historical facts regarding the trial and then propose some of the potential causes for this failure, hoping to discover where things went wrong.

Perhaps some methodological choices were ill-advised and we will attempt to identify ways that the trial could have been more successful. Research that questions the merit of interventions that are currently offered to many patients but that remain of unproven benefit will always be difficult, but if we want to practice a scientific medicine in the best interests of patients this is exactly what should be done. In a last section, we will propose how this aim could be achieved: by recognizing a special status for this type of 'clinical care research'.

### The TEAM trial

Endovascular treatment (EVT) with detachable coils has been a treatment option for intracranial aneurysms (IAs) since 1991. There is no dispute that *ruptured *aneurysms (RIAs) need to be treated if we are to prevent re-ruptures. A trial on RIAs, comparing surgical clipping and endovascular coiling (ISAT), started as a pilot study in 1994. It ceased recruitment in 2002 after enrolling 2143 patients. ISAT showed better clinical outcomes at one year for patients treated with coiling [1,2]. However results of ISAT cannot be applied to unruptured aneurysms (UIAs) [3]. With the use and availability of non-invasive neuroimaging, particularly MRI, UIAs are increasingly discovered as incidental findings and coiling of UIAs has become the most frequent neuro-endovascular treatment performed in many centres [4]. A RCT on treatment options for UIAs has never been done; most clinicians and patients who have resorted to preventive clipping or coiling of UIAs have done so on the basis of fear of ruptures and purported efficacy in RIAs [3]. Because UIAs are much more frequent than RIAs (approximately 1-2% of the population as compared to 10/100 000) and because the hemorrhagic risks of UIAs are much lower than the risks of re-rupture of RIAs (1% per year compared to 30-50% within the first year), the main question, regarding UIAs, is not whether one treatment option is better than another, but whether any risky preventive treatment is justified. An earlier international effort to register the results of treatments and observation in 4060 patients recruited between 1991 and 1999 [5] suggested that treatment was rarely justified and proposed 5 year estimates of the risks of rupture for lesions of various sizes and locations, but the study was fraught with all the pitfalls of an uncontrolled observational study [6]. There are reasons to believe that coiling is initially less morbid than clipping, but the long term efficacy of coiling in prevention of bleeding has yet to be shown [7,8]. Hence the main problem with coiling of UIAs is that while the intervention is frequently performed, nobody knows whether patients have better clinical outcomes with coiling or observation. The TEAM trial was designed to answer this specific question [9-11]. The objective of TEAM was to recruit 2000 patients with UIAs in 40-60 international centres within 3-4 years. The planned follow-up period was 10 years.

A calendar of selected events is shown in Table 1.

**Table 1:** Calendar of selected events

**Date**	**Event**
2000-2003	Discussion with peers regarding details of trial design
2004	Initial submission to NINDS and CIHR
Sept 2004	Publication of protocol, version 1 (11)
Feb 2006	Conditional approval by CIHR
Feb-Jun 2006	DSMC Charter
Jun 2006	Official approval for a third of support
Sept 2006	Full financial support
	Application to IRBs for trial initiation
May 2007	Approval for French Centres
June 2008	Approval for UK Centres
July 2008	Publication of final protocol in Trials (10)
Sept 2008	Letter of progress to CIHR
	Application to NINDS for US Centres
Oct 31^st ^2008	Unilateral trial interruption ordered by CIHR
November 6^th ^2008	First International investigator meeting
June 2009	Trial Interruption by Steering Committee
2010-2011	Preparation and publication of final report

The first version of the proposed protocol was published in September 2004 [11]. Subsequent discussions with the CIHR for 2 years led to minor protocol modifications that, given the ultimate fate of the trial, can be judged inconsequential. In the meantime an invitation to submit to the NINDS was, after consultation with its officers, and given the CIHR intent, declined by investigators. The CIHR ultimately approved the protocol in February 2006, but requested that the Data Safety and Monitoring Committee submit a charter with predefined stopping rules before issuing a final decision. Support was officially granted in June 2006, but the CIHR offered 30% of the budget requested. The investigators claimed that such a large scale effort could not be launched without some assurance that resources would be sufficient to give it a good try and intensive negotiations over the summer months led to full financial support for 5 years in September 2006 (approximately $5 million for 5 years, a budget felt to be insufficient by a factor of 3-6 by most clinical research organizers). Trial coordination was to be performed in 2 centres: Oxford for European and Montreal for North-American sites. In 2006, the P.I. of the Oxford centre applied for financial support at the UK National Institute of Health Research (NIHR) Health Technology Assessment Panel (HTA) for additional support, which took one more year, but was successful [12]. The ultimate version of the TEAM protocol was finally published in 2008 [10]. Collaborating US physicians applied in 2008 to the NINDS for complementary support of a national coordinating centre to encourage U.S. participation. This would ultimately be refused, after CIHR interrupted funding in 2008. A small grant was also obtained by a centre in Brazil in 2008.

The CIHR had a non-voting representative at the Steering Committee, but the DSMC was composed of fully independent, voluntary members using the framework published by the DAMOCLES group [13].

Although letters of intent had been provided by more than 30 investigators in 25 centres as early as 2004, the official applications to local, regional, national Committees could not be initiated before September 2006. Official approval by all authorities necessitated between 6 months (in French and Canadian sites) and 2 years (for ethical committees and the UK Hospital's Research Governance departments). These delays, although excessive by any standard, are nowadays routine [14].

The first international investigator meeting was planned to occur in Amsterdam Thursday November 6^th ^2008, but on Friday October 31^st ^at 16h00 the coordinating centre in Montreal received an email from the CIHR scientific officer ordering, without any prior notice or discussion with the Steering or Data Monitoring Committees, interruption of the trial as of October 31^st ^2008. The CIHR decision, made after consultation with a secret, anonymous peer-review committee, we were told, was based on an interim report (September 2008) showing insufficient recruitment of patients.

It was too late to cancel the Amsterdam meeting, where participants were keen to continue recruitment. A Steering Committee meeting on December 4^th^, 2008, voted for continuation of recruitment until a response to our request for revision of the CIHR decision, and until results of other applications were known. Discussions regarding trial continuation despite interruption of funding can be found in reference [15]. Appeal of the CIHR decision was refused and in view of withdrawal of funding, the additional support from the NINDS was denied. The trial was officially stopped June 28^th ^2009. By that date, 50 centres were registered and 80 subjects had been recruited. This poor performance can hardly be explained by a lack of visibility: Between 2004 and 2009, the trial had been presented at 18 annual meetings of 9 different major professional international associations (sometimes repeatedly), at 25 annual meeting of 19 national associations, and at 20 Grand Rounds of various participating centres. In each country a collaborator was responsible for discussing TEAM at all possible regional or national meetings. Two press conferences in Europe and North America led to articles in 36 different magazines and newspapers, sometimes on the front page of major public newspapers. The TEAM collaborative group published 21 manuscripts related to various aspects of the trial, scientific and ethical concerns, and reviews on unruptured aneurysms in peer-reviewed journals between 2004 and 2010.

**Figure 1:**
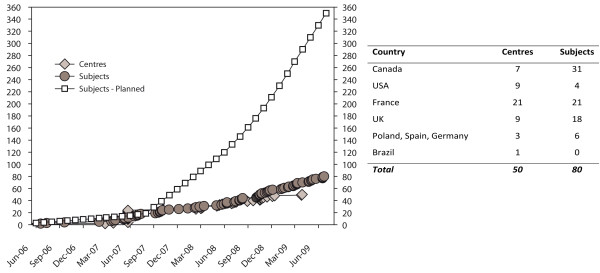
**Centre and subject accrual in the TEAM trial, from June 2006 to June 2009**. An estimate of expected subject accrual is given for centres having contributed at least one subject, based on a rate of one subject/per month/per centre.

Poor recruitment combined a) severe delays in trial initiation mainly caused by bureaucratic barriers in many countries and institutions; b) low recruitment rates even in those centres that did initiate the trial, caused by a reluctance of participating physicians to recruit all or most eligible patients, and by patients' refusal to participate in many cases. For example, a survey performed at the first recruiting site showed that the trial was proposed to 55% of eligible patients, but only 18% of patients that were approached agreed to participate. Figure 1 show the time course of centre and actual as well as projected subject accrual, and their distribution by country. Table 2 gives baseline data on randomisation as well as number of outcome events (0) and mean duration of follow-up. There was one peri-procedural complication (a brachial hematoma), but no disease or treatment-related neurological event in either endovascular or conservative management groups.

**Table 2:** Baseline data on randomization, number of outcome events and mean duration of Follow-up

	EVT	CT	Total
	**N = 42**	**N = 38**	**N = 80**

**Age - yr**			

**Mean (SD)**	**56 (10)**	**54 (10)**	**56 (10)**

**Range**	**34 - 73**	**26 - 78**	**26 - 78**

			

**Gender - %**			

**Female**	**60**	**71**	**65**

**Male**	**40**	**29**	**35**

			

**Number of unruptured aneurysms (untreated) - %**			

**1**	**83**	**89**	**86**

**2**	**14**	**8**	**11**

			

**History of SAH - %**			

**Yes**	**14**	**5**	**10**

**No**	**86**	**95**	**90**

			

**Target aneurysm size - mm**			

**Mean (SD)**	**6 (2)**	**7 (3)**	**7 (3)**

**Range**	**3 - 11**	**3 - 16**	**3 - 16**

			

**Target aneurysm location - %**			

**Posterior**	**5**	**13**	**9**

**Anterior**	**95**	**87**	**91**

Ophthalmic region	35	30	32

Middle Cerebral artery			

	25	30	29

(% for anterior normalized to 100%)			

			

**Outcome events**			

**Number**	**0**	**0**	**0**

			

**Duration of follow-up - months**			

**Mean (SD)**	**14.6 (11.0)**	**12.6 (9.4)**	**13.6 (10.2)**

EVT = Endovascular therapy; CT = Conservative treatment

## Discussion

The failure of the TEAM trial is multi-factorial. All potential causes cannot be reviewed here.

Before addressing some of the potentially generalizable causes for the premature interruption of this particular trial, causes that may be pertinent to other clinical endeavours, the senior authors, who are primarily clinicians rather than professional trialists, take full responsibility for the end-result. We could perhaps have done a better job at promoting TEAM. Although most clinicians of the field acknowledged the necessity for doing the trial, formal barriers and cultural resistances were so numerous and entrenched that many thought the entire enterprise was 'ideal but, given the current clinical environment, bound to fail'. Although this experience may be used as an example of what not to do if the ultimate goal is a successful research career, the present article was rather written to explore what could be attempted to make a *necessary trial *a clinical reality for the benefit of present patients.

We first discuss some of the problems with the design and some of the obstacles that were encountered. For each problem, a piecemeal solution will be suggested, as well as a global, revolutionary one at the end of this review. As we experience these formidable difficulties, the reader should keep in mind that the two treatment options TEAM was proposing were entirely standard ways of managing patients with UIAs, in day to day use in all centres. The only difference from standard care was that there would be i) randomized allocation of treatment in the minority of patients willing to participate and ii) centralized web-based collection of simple anonymous data on follow-up visits that are part of normal clinical routine.

### Factors linked to the design of the trial

#### a) Sceptical versus enthusiastic trials

While all trials are built on hypotheses that must be scientifically tested in the real world, some trials have a power of seduction that others do not have. Some trials fuel the hope that in a bright near future a new approach, device or treatment will provide a chance to conquer new territories. For participating patients this may mean hope for a cure or a clinical improvement when it was not possible before. For physicians the trial may carry promises of new powers to help their patients or to control a disease. This first kind of trials could be called 'enthusiastic'. Other trials like Team are necessary because a practice is increasingly used while nobody knows if it is doing good or harm. This other type of trial could be qualified as 'sceptical', because it does not promise a novelty; it specifically asks for a rigorous evaluation of the true benefit, if there is any, of an intervention people have already access to; hence it can only draw on a sense of prudence and duty, imperatives that are less 'transporting' than hope or enthusiasm. This distinction grossly corresponds to 2 diverging roles of science: scientific research as a platform for projections into a promising future, and science as a normative framework to rigorously assess present actions. Marketing of the trial to patients and recruitment could have been easier if we could have claimed 'stand up to aneurysms, the silent killer' the way some claim 'stand up to cancer'. This would call for a very different trial, a trial on the benefit of *screening *for UIAs. Since the trial questioned from the outset if therapy was beneficial, we could not launch large scale screening campaigns, even if we had the resources, to alarm a large number of healthy individuals with incidental findings, and to propose a potentially futile, perhaps harmful fight against an asymptomatic disease they did not know that they had. Although we were aware some clinical research in prostatic cancer or aortic aneurysms had taken such a path [16,17], our aim was to assess if therapy, as currently used in patients with incidental findings, was effective and beneficial. Perhaps in the future a trial on UIAs could be more easily completed if it was combined with a trial assessing the benefit of screening, but such an endeavour carries a much greater risk of iatrogenia at a large scale. We remain reluctant, however, to promote the success of a clinical trial by first instilling fear to recruit patients, to later attempt to defuse it and reveal how uncertain the benefits of therapy are. Hence this type of research can only draw on a notion of prudence and rigor. But scientific rigor and caution in proposing elegant but risky preventive interventions are much harder to sell than enthusiasm and rosy perspectives of the future. This first problem may be difficult to circumvent, for there may be no easy or popular way of insisting on scientific methods and intellectual rigor in human behaviour. Only an ethical imperative could possibly do the trick (see ethical issues below).

#### b) Loser trials versus Winner trials

A trial becomes more difficult to complete when it does not provide some kind of concrete incentive to clinician-investigators ('what's in it for us?'). Endovascular techniques are elegant, elective, fashionable and in many countries lucrative for physicians and institutions. Even when physician or institution income does not depend on the number of treated patients, increased case volume is desirable. The reputation of the centre or of doctors, the designation of the institution as a referral centre (with the correspondingly larger budgets), increased ability to recruit colleagues, and even physician credentialing (for example in France or in Japan which require a minimum number of interventions), all forces support the notion that greater case volume is better, if not for patients, at least for care providers. Specialists performing EVT, like most surgeons, actually enjoy their work, and questioning the value of their interventions is unlikely to be popular. This problem occurs in most 'operate-don't-operate' surgical trials. Success would have been easier to achieve if we could guarantee that the trial would bring more patients to endovascular clinics, instead of the perception that the trial would potentially decrease their activity by 50%. This reality led Houdart [18] to distinguish 'winner' trials, trials that could lead to a gain for the clinicians doing the investigation (any gain, whether in income, clinical activities, turf battles), from loser trials, such as TEAM. One way to turn such a trial into a 'winner trial' is to make reimbursements for the unproved interventions dependent on participation in the trial. This suggestion, previously helpful in at least one centre in the ISAT trial, seems to be verified with the recent success of SAMMPRIS [19]. Although we suspect that in the presence of fear, naive faith in technology, and unreliable knowledge, autonomous decision making is precarious, this coercive proposal is bound to be controversial, with concerns that revolve around justifications to limit physicians' and patients' free autonomous choices.

#### c) The choice of the comparator intervention

The contrast between the two arms of the trial (active versus conservative management) may have been simply too marked to be palatable to physicians and patients. Although both arms were to be clinically followed in the same manner, with conservative management of risks factors (such as smoking cessation, and control of hypertension if present), and follow-up imaging as prescribed by each centre, patients often felt the choices were between being cared for and being denied care. One solution here is to offer a drug or a placebo (even though no such therapy currently exists) to support the hope for being 'treated' in some sort of way. The other benefit to inclusion of a placebo group may be to help patients understand and believe that the 'natural history' of the disease is not as bad as they may initially think, and that the appropriate intervention must be correspondingly very safe, to the point that a placebo may be appropriate, if we are to prevent large scale iatrogenic damage to patients.

Another alternative would be to offer regular follow-up imaging, although this is an expensive management strategy; repeated non-invasive imaging studies are costly, especially if they are repeated in a yearly fashion for thousands of patients, and are themselves of unproven value. Such close imaging monitoring may be falsely reassuring, since patients may still bleed between studies, or falsely alarming, since no one has shown that even aneurysms that have enlarged must be treated.

#### d) Randomization methods

Because many patients who were offered participation were already convinced something must be done and because physicians were reluctant to question the merit of their intervention, we could have resorted to asymmetrical allocation of management, such as 2:1 or 3:1 in favour of treatment, as in some other interventional trials [20]. Of course the number of patients to be recruited must then be increased; more importantly, this option gives the false impression that we know active treatment is superior. If treatment turns out to be harmful, every recruited patient, at the time of enrolment, has been subjected to a larger risk, when compared to 1:1 randomization.

Another method that was explored was a modified Zelen trial, with pre-consent randomized allocation to treatment groups, a method that had previously saved difficult breast cancer trials [21,22]. A major protocol modification would have necessitated protracted delays in an already obstructed trial. The use of this method has been controversial [23-26] but we believe it must be seriously reconsidered if we want to somehow counterbalance prejudgment, wishful thinking, the illusion of knowledge or control, conflicts of interest and the cultural resistances to necessary trials.

#### e) Uncertainty versus pseudo-knowledge

The trial was conceived as a 'management' or 'pragmatic' type of trial, with inclusion of any patient eligible for prophylactic coiling. Many physicians would have preferred more precise directives, and more narrow selection criteria. Some would have restricted the trial to low-risk lesions (< 7 mm anterior circulation aneurysms for example), taking for granted the value of treatment in higher risk patients. At the same time many others would have excluded the same low-risk patients, claiming they could but minimally benefit from a prophylactic intervention and could only decrease the potential for showing treatment in a favourable light. In the absence of reliable data and since both small and large aneurysms were being treated in most centres, the recruitment of individual patients was left to the clinical judgement and 'equipoise' of treating physicians. It is worth noting that the mean size of aneurysms in patients recruited in Team was exactly 7mm, the supposed threshold for risk of rupture [27](Table 2). Future trials on UIAs may have to consider beliefs of the community, or the reassurance provided by arbitrary limits, more seriously, no matter how weak the evidence.

The danger of course is that arbitrary limits gain credibility and is acted upon, both inside and outside the trial, without scientific justification (see [16] for an example of an arbitrary size limit to prescribe interventions in AAA).

#### f) The investigators

The Team trial required the same physicians performing the interventions to question the value of their practice. This easily leads to conflicts of interest, as discussed above. One difficulty specific to the trial was that in some countries and institutions, neurosurgeons not practicing endovascular treatments were the primary clinical decision makers, to whom patients with UIAs would be referred, whilst interventionists participating in the trial were secondarily consulted on endovascular management of these patients. Neurosurgeons formed a view whether treatment was warranted, and then if aneurysms should be clipped or coiled. Once they referred patients for coiling, a commitment to treatment became almost irreversible in the minds of the clinician and patient. Perhaps other physicians should have been involved, such as neurologists, who have a better track record at successfully completing trials. Being less directly concerned by the merit of the interventions, they could also have provided more objective information to patients. Unfortunately at the present time neurologists are infrequently involved in the management of UIAs, a situation that could change if some medical or pharmacological treatment was explored, in a 2 × 2 factorial design, for example. Another potential solution could have been to include a surgical arm to the trial, to attract the interest of vascular neurosurgeons, but this would have added another element of complexity to trials aimed at finding the best management of aneurysms [9]. A trial comparing surgical and endovascular management of UIAs has recently been launched [28].

#### g) Investigator-based trials versus trials sponsored by the Industry

In some respect the fact that the trial was not sponsored by the Industry may have reassured some patients that are suspicious about conflicts of interests and hidden motivations behind trials. In other respects it made a trial deprived of the market forces and financial power of multinational companies less credible, at least to some IRB members, some legal or national regulatory offices. Who would be responsible for expenses, for complications, who would respond to lawsuits? Is the enterprise strong enough to support its ambitious goals? The fate of TEAM is an empirical proof that their doubts were realistic, of course. TEAM did not have sufficient resources to resort to contract research organizations, even those that are university-based, but given the specialized nature of the intervention, it is unlikely that such an organization would have had more success.

### Factors related to legal and bureaucratic hurdles

The list of problems, conflicts and delays related to diverging or contradictory rules and regulations throughout various countries and institutions is simply too long to be considered here; it has been extensively documented before [29] but a few points deserve attention. Bureaucratic hurdles cannot be held directly responsible for poor or slow recruitment, but they certainly contribute to excessive delays in initiating trials. Up to 2 years (in United Kingdom) were necessary to complete this process, where centres had just barely been approved when financial support was withdrawn. This may adversely affect the motivation of collaborators and the momentum of potential trial participants. As things evolve towards ever more stringent and rigid regulation, research efforts will progressively be restricted to profit-oriented enterprises led by the Industry. The irony is that the rules and regulations were not designed to obstruct academic studies or clinically pertinent research questions. Often, when reviewing regulatory documents, it was impossible to identify the clauses that applied to those pragmatic academic trials designed to test management strategies in day to day clinical use. The existence of this type of trial seemed to have been forgotten when the legislation was created [30].

In many countries, changes designed to provide harmonization (throughout the European Union for example), were ongoing and only partially successful. The new rules were still in the process of being interpreted and understood at the same time we were seeking approval [31]. This led to contradictory and sometimes erroneous advice and requests from various authorities in diverse institutional or national offices. Sometimes no one knew what to do. Some offices could not figure out how to fill out their own forms. Who is the 'Sponsor' of an international academic trial? Funding agencies cannot act as 'sponsors' and various legal consequences were linked to this nomination. We can only hope for real, in-depth harmonization if pragmatic international trials are to become feasible. National research institutes should engage in multilateral collaborations, to insure that their rules do not contradict each other and that RCTs addressing current clinical dilemmas are not systematically obstructed. For example, CIHR rules that forbid overhead charges on transfer payments to other research centres, insurance costs for enrolled patients, and up-front fees for IRB reviews, contradicted rules in the UK, France and USA, which mandate overheads for the Oxford Coordinating centre, the requirement for special insurance to cover patients recruited in France, and the frequent requests from US centres for a $3-5000 dollar up-front fee to examine the TEAM protocol. When harmonization is not yet possible, then perhaps the institute providing the financial support to the trial should be able to relax some of its own internal rules to help international efforts in dealing with other countries' requirements.

Something must be said about research contracts. At each institution a legal office, trying to provide maximum protection for the institution and their doctors according to national laws, and to ensure research was going to proceed 'the right way', tried to impose its own local clauses. No matter how often we reminded people that TEAM was simply a test of currently used treatments, with randomized allocation and anonymous web-based reporting of clinical outcomes; no matter how minimal the monetary compensation TEAM provided to participating sites, most institutions (including our own) insisted on negotiating contracts that were supposed to reconcile all the legal diversity of the world with zero risk tolerance. This of course is costly, time consuming and illusory. Is this really protecting patients? When one considers that most centres recruited between 0 and 3 patients, these precautions were indeed excessive and completely counterproductive.

More importantly, bureaucratic obstacles and the time spent to overcome them now appear to represent a major reason why clinicians consider clinical trials an inaccessible, indeed illusive means to address important clinical dilemmas. In some specialties like neurovascular interventions, clinical research mostly consists in case series and registries, and very rare trials. There is even a recent trend to replace trials with large data bases and powerful computers [32]. But how could recording our day-to-day actions protect the very same patients that are subjected to these treatments, which have never been validated as beneficial? As things stand, randomized trials cannot become a meaningful part of clinicians' work and responsibilities; until this is corrected trials will remain outside the culture of main stream patient care where they should be.

### Marketing of the trial

We have consulted 2 private and 2 University-based experts on marketing, an aspect of the promotion of clinical trials that is gaining popularity in its own right [33]. The marketing challenges involved in studies like Team are simply formidable. This problem is related to the sceptical nature of the research question (see above). We did not consult patient support groups or lay persons in the design of the trial, however. Regarding the preparation of information booklets and consent form, we abandoned many efforts at promoting this material after they had been repeatedly rejected by IRBs as 'too biased in favour of participation'. For example, a frequently rejected sentence mentioned that 'given the present uncertainty, your physician believes that the best option is to participate in the trial'. It seems that many powerful people still believe that guessing (usually in favour of intervention) is the best treatment that should be offered to the patients of their institution.

### Financial obstacles

The level of financial compensation per patient offered to participating centres (mean $800 Can) was nowhere near what is usually offered by Industry or even some NIH-funded trials. It is questionable however if this factor alone had a large impact on the trial. Other important financial issues included the fear of seeing reimbursement for the treatment denied by insurance companies in certain countries, or of a reduction in income for physicians or institutions. In contrast, in other countries where EVT of UIAs was not so common, institutions feared an explosion of costs for devices, hospital stays and procedures. If money were really a pertinent issue, vast amounts would be necessary to compensate for costs of devices and procedures, complications, potential losses in income etc... The logical source for such a large amount of money would be to look at those that have vested interests in the results of the trial: device companies, health care providers and insurance companies. However repeated attempts to secure financial support from the Industry failed. Unlike pharmaceuticals, many medical devices are approved without randomized clinical trials. In fact, all neurovascular devices approved in the last 3 decades have been introduced with registries of 100 or fewer cases, without controls. It seems that our field is in no need for objective appraisal of the value of our interventions. The involvement of health providers, private or public, is an option that could be perceived with suspicion because there could be a conflict of interest. Trials like TEAM are not protected from conflicts of interest, however, and support from a public agency is obviously not a sure way to secure completion of a difficult but necessary trial. Research projects compete for scarce resources and an eagerness to redirect dollars to 'more promising' research endeavours is always a threat.

Ways to obtain modest support for a feasibility or a start-up phase (from local charities, local research funds, etc.) exist, and have been successful before, with larger sums being released by agencies once feasibility has been shown [34-37]. This path may reduce the number of years necessary to the launching of a trial like Team. One problem is that as soon as money is involved, contracts are usually required. In addition, we believe that the 'feasibility' notion is a non-scientific, circular notion, susceptible to endanger the trial feasibility itself: what can we conclude from the failure of a modest, local, unfunded attempt to initiate a 2000 patient, international clinical research duty? [15].

Some have claimed that the only way trials like TEAM would be successful would be to condition reimbursement of interventional procedures on participation in the trial. Of course this controversial proposal raises ethical and societal issues that are beyond the scope of this article [38].

More fundamentally the cost issue is vitiated by misconceiving what this type of clinical research, which addresses the value of current management strategies, is doing, as opposed to research aiming at the discovery of some future promising treatments. Where care which costs ten times more is already covered, why should physicians and institutions wait for more money in order to assess whether they are doing good or harm? [15]

### Cultural factors

All the foregoing difficulties contributed to delay and obstruct the trial, but we still have not covered the main problem. Ultimately the major obstacle to recruitment is a clinical culture, equally shared by physicians and patients, which demands of physicians to know what to do, whatever the circumstances. There is no room for the unknown or the uncertain. As clinicians we are trained to perform actions in a repetitive fashion. When confronted with the uncertainty, our tendency is to cut inquiry as short as possible, to make the speediest return to actions we have been trained to perform. We learn that such actions should be individualized to each particular patient. We are trained and certified to believe that we know what to do, even when we do not. Our unjustified confidence finds resonance in patients, who hate hearing from their doctors "I do not know". In a clinical world where research is excluded, suspension of judgment cannot exist. For each patient corresponds a (felt) most-appropriate action that then, whether correct or not, becomes mandatory. We have completed a circle: in the absence of trials, one must choose a single best option in each case; once one is trained and expected to find a best option in each case, trials become difficult if not impossible. This culture is reinforced by a research-care dichotomy that automatically makes research suspect and optional, while care is a necessity. "Practice'', according to the Belmont report, refers 'to interventions that are designed solely to enhance the well-being of an individual patient and that have a reasonable expectation of success', while 'research', now divorced from 'practice', is defined as 'an activity designed to test an hypothesis, permit conclusions to be drawn, and thereby to develop or contribute to generalizable knowledge' [39]. This is as good a definition of science as can be. But how could we accept to condemn medical care to an unscientific practice? This culture is so natural, so entrenched, that it has led to the exclusion of scientific and research methods from routine clinical care, an exclusion that has not been shocking to most people. This circle can only be broken by an ethical imperative: medicine should use interventions that have been proven beneficial; for clinicians proposing unproven interventions, treatment may be offered, when clinical judgment indicates, but only within the context of an RCT. Perhaps we can reconcile everybody by requiring that clinical judgement lead to 2 (instead of 1) favoured management strategies: a clinical trial comparing these 2 options.

### Ethical issues

The failure of the TEAM trial is an opportunity to expose a fundamental problem that plagues modern medicine, a problem which may explain why this type of clinical research is most of the time not even attempted, ultimately with grave consequences for patients: the research-care dichotomy. Forged in the aftermath of the research scandals of the mid-twentieth century [40], the divorce between care and research deprives clinical medicine of its science and condemns physicians to practice an unverifiable medicine founded on beliefs, opinions, intentions and intuitions rather than on validated patient outcomes. Upon careful examination, most cultural, conceptual and bureaucratic obstacles that obstruct trials integrated to clinical care draw on a one-sided, biased view of the role of research in medicine. According to this view, research is an intruder in clinical care, an enterprise dedicated to the benefit of future patients, a source of potential conflicts of interests that must be controlled. This view misses the truth-seeking and truth-preserving normative role of research methods essential in defining what good clinical care should be, immediately, for the benefit of the present patients. The Helsinki Declaration is more balanced and clearly prescribes a duty of research when reliable evidence is in wont: 'The primary purpose of medical research involving human subjects is to ... improve preventive, diagnostic and therapeutic interventions (methods, procedures and treatments). Even the best current interventions must be evaluated continually through research for their safety, effectiveness, efficiency, accessibility and quality.' (Declaration of Helsinki clause 7 [41]). 'In the treatment of a patient, where proven interventions do not exist or have been ineffective, the physician...may use an unproven intervention ... Where possible, this intervention should be made the object of research, designed to evaluate its safety and efficacy.' (Declaration of Helsinki clause 35 [41])

Instead of obstructing trials that aim to define what a good practice could be, in the name of an ethics of clinical research, we need a more inclusive ethics of clinical care that formally prescribes such trials, for the sake of protecting all patients, especially those currently confronted with a clinical dilemma, and otherwise subjected to the intervention in need of validation. Modern medicine needs an account of the ethics of clinical care that acknowledges the current limitations and risks of medical interventions, the existence of alternative courses of action, and the necessity for verification of purported benefits, up front and in a transparent manner: medicine needs to reintegrate scientific methods into medical care, and an institutional and bureaucratic system that encourages, rather than obstruct, such a pursuit of truth in defining a good medical practice.

### A revolutionary option

TEAM questioned whether preventive coiling was doing more good than harm. Its failure may be an occasion to question whether all conceptual and bureaucratic obstacles that have been devised and implemented in the name of ethical and research governance are not themselves doing more harm than good. We can only provide here the canvas of a more global solution. The objective of clinical research is to prevent errors; errors in medicine translate into unnecessary morbidity and mortality. The crux of the matter should not be to limit the intrusion of science into medical care but how to properly integrate clinical research and care that truly benefits present patients. 'Clinical care trials ' (CCTs) are necessary to offer an alternative to current unverifiable medical practices and to counter the non-sensical idea that good clinical care could be provided outside science or that a good medical practice could be defined with studies performed outside clinical care. Scientific methods can provide norms to protect patients from interventions that have yet to be proven beneficial. The ethics of clinical care research can be founded on a principle of caution: either physicians propose validated interventions, or they propose promising interventions only within controlled trials. The notions of clinical equipoise need to be replaced by a notion of asymmetrical uncertainty, with a duty of research when the contemplated action has not been validated before. The issue cannot be a fragile equilibrium, which of our unjustified beliefs wins the battle whether at the level of the individual or of the community of experts. The notion of therapeutic obligation (which transpires through most interpretations of equipoise) must be revised [42]. Currently, when confronted by the uncertainty, therapeutic obligation proposes the following maxim: 'When in doubt, indulge into believing that you know, that you are good, act and get paid.' An 'obligation' has never been easier, no wonder it is so popular! The ethical obligation, of course, is in the other direction. The main issue regards the ethics of physicians' beliefs and actions: we must require that beliefs be founded on rigorous evidence to justify potentially risky preventive actions. Hence we must start with an ethical imperative not to act pretending we know when we do not, but to acknowledge that our treatment preferences are based on hypotheses that need to be tested. The intervention can then be offered, but with an equal chance of escaping false promises and be treated by a validated alternative, by using randomization. If research provides normative methods to care in the presence of uncertainty, the care of present patients provides rules to the design of clinical care trials. These can be developed as large pragmatic trials, comparing unproved interventions with a validated alternative (or conservative management, when none exists), with simple, meaningful clinical endpoints, and no extra test or risk beyond what is considered normal care. This type of trial is not new [43-48]. The emphasis in 'pragmatic' or 'management' types of trials has been on providing answers that are applicable to the real world, most pertinent to policy makers [49]. This time, with CCTs, the emphasis is on protecting current patients confronted with a clinical problem. Hence the doing the trial is a good in itself, a primary good that does not depend on the final, scientific results. The fact that what is best for current patients confronted with the uncertainty is also what will turn out best for decision making is not fortuitous, of course. As long as the type of care that is being trialed is already reimbursed, and if there is no or minimal interference with care, no added test and no extra risk, there is no need for financial compensations, no need for separate funding, for time-consuming contracts, for legal or bureaucratic pestering that will inevitably interfere with the goal of the trial: to help physicians provide prudent care in a context where evidence is lacking. It is possible that what is needed today was achievable decades ago [50] but has become impossible. One of us believes that the ISAT trial, a turning point in our field, could no longer be realized in today's world [1,2]. Is this progress or regress?

The role of public agencies and clinical care research governance needs fundamental redefinition. The default position of the agency should be to support the principle that this type of clinical trials is not a luxury, but a necessity. To avoid a self-defeating process, peer-review cannot be a competition between trials that are necessary to the care of present patients. It should serve as a consultation table to provide expert advice to improve proposed clinical care research. Institutions should provide fast-track examination of clinical care trials free of charge: they are essential to a good practice. The importance of clinical care research should be taught at all levels (students, patients, institutions, local, national and international committees), to promote the cultural revolution that will make these trials the gold standard of care in the presence of uncertainty. Ultimately any system which delays and obstructs ethical research of treatments which are current and widespread use but lack scientific randomised evidence should be revised.

### What are Clinical Care Trials (CCTs)?

The adjective 'revolutionary' (as in 'a revolutionary option') is in one sense an exaggeration, since the scientific methodology already exists, in another, the term is a close estimate of the magnitude of what is needed to overthrow the current obstacles to clinical care research. The label intends to emphasize that the CCT is needed to properly care for patients. This is not the place to fully define what clinical care trials could be, but we can broadly brush some fundamental characteristics: CCTs offer the possibility of using medical interventions that, according to current beliefs, or perhaps some pathophysiological reasoning, seems promising, but that have until now never been validated as beneficial. At the same time the trial protects patients from what may potentially sway their choices and their physicians: false promises, fashion, marketing, corporate or wishful thinking. Treatment options are available and are in current clinical use. The design of the trial does not include tests or actions that are not necessary to the safety or the care of present patients confronted with the dilemma. Selection criteria are minimal, because the trial offers a way out of the dilemma to all or most patients in need. Patients are not used to show treatment in a good light, to forward Science or Knowledge for future patients; rather, scientific methods are used to protect present patients from the illusion of knowledge and extraneous forces and interests. Hence there is no conflict between the interest of the present patients and the knowledge that may serve future patients and no possible 'therapeutic misconception' [51]. An important secondary benefit is that there is no extra cost or personal beyond what is necessary to care for these patients. Institutions and physicians participate, without requiring extra monetary compensation, because it is the best medical care they can offer in the presence of uncertainty.

## Conclusion

Trials like TEAM will remain extremely difficult, but they will become impossible if the current trends towards an explosive bureaucracy are not reversed. A special category for this type of trials should be created, and the process for implementing clinical care trials greatly facilitated, if the community of clinicians is to be able to correctly identify what could be a good medical practice.

## List of abbreviations used

TEAM: Trial on Endovascular Aneurysm Management; EVT: Endovascular Treatment; IA: Intracranial Aneurysm; RIA: Ruptured Intracranial Aneurysm; UIA: Unruptured Intracranial Aneurysm; RCT: Randomized Controlled Trial; P.I.: Principal Investigator; CIHR: Canadian Institutes of Health Research; NINDS: National Institute of Neurological Disorders and Stroke; DSMC: Data Safety and Monitoring Committee; NIH: National Institute of Health.

## Appendix

### TEAM Collaborative group

#### Steering committee

Pr Jacques Moret, Paris; Dr Alejandro Berenstein, New York; Dr Herman Zeumer/Jens Fiehler, Hamburg; Dr In Sup Choi, Boston; Dr Cameron McDougall, Phoenix; Dr Gabriel J. E. Rinkel, Utrecht; Pr Ling Feng, Beijing; Dr Julian Spears, Toronto; Dr Jean Raymond, Montreal; Dr Andrew Molyneux, Oxford; Dr S. Claiborne Johnston, San Francisco; Dr Isabelle Rouleau, Montreal; Dr Allan J. Fox, Toronto; Dr Jean-Paul Collet, Vancouver; Dr Yves Lepage, Montreal; Antonieta Gasparini (CIHR, Ottawa); Guylaine Gevry, Ruby Klink and Marcia Loor, Montreal.

#### Data Safety and Monitoring Committee

Pr Luc Picard, Nancy (Chair); Dr Michael Eliasziw, Calgary (clinical statistician); Dr Louise-Hélène Lebrun, Montreal (neurologist); Dr Gerald R. Winslow, Loma Linda (ethician); M. James Hosinec, Montreal (patient representative).

#### Clinical Events Committee

Dr Charles Strother, Madison (Chair); Dr Karl-Fredrik Lindegaard, Oslo (neurosurgeon); Dr Daniel Roy, Montreal (neuroradiologist); Dr Sylvain Lanthier, Montreal (neurologist).

#### EndPoint Review Committee

Dr Robert Coté, Montreal (neurologist); Dr Jeffrey Minuk, Montreal (neurologist);

Dr Ariane Mackey, Quebec (neuroradiologist).

#### Expert Committees

**Imaging Center**: Dr Allan J. Fox, Toronto; Dr Alain Weill, Montreal

**Data Preparation and Masking Center**: Dr Philip White, Edimburg

**Neuropsychology**: Dr Isabelle Rouleau, Montreal

**Patient Support Group**: Dr Maria Angeles de Miquel, Barcelona

### Participating centres

#### France

Angers Hôpital Larrey (Pasco-Papon A.); Besançon CHU Jean Minjoz (Bonneville J.F.); Caen CHU Côte-de-Nacre (Courtheoux P.); Clermont-Ferrand Hôpital Gabriel Montpied (Chabert E.); Colmar Hôpital Pasteur (Tournade A.); Créteil Hôpital Henri Mondor (Gaston A., Blanc R.);

Grenoble Hôpital Albert Michalon (Le Bas JF.); Lille Hôpital Salengro (Pruvo J.P., Leclerc X.); Limoges Hôpital Dupuytren (Chapot R.); Lyon Hôpital Pierre Wertheimer (Turjman F., Lamy B., Tahon F.); Nancy Hôpital Central (Bracard S., Anxionnat R.); Nantes Hôpital Laennec (De Kersaint Gilly A., Desal H.); Paris CH Sainte-Anne (Meder J.F., Trystram D., Godon-Hardy S.); Paris Fondation Rothschild (Moret J., Piotin M., Spelle L., Mounayer C.); Paris Hôpital Saint-Joseph (Zuber M.); Paris Hôpital Lariboisière (Houdart E.); Paris Hôpital Pitié-Salpêtrière (Biondi A., Bonneville F., Jean B., Sourour N., Chiras J.); Reims Hôpital Maison Blanche (Pierot L., Gallas S.); Saint-Etienne Hôpital Bellevue (Manera L.); Suresnes Hôpital Foch (Rodesch G.); Toulouse Hôpital Purpan (Cognard C., Januel A.C., Tall P.); Tours Hôpital Bretonneau (Herbreteau D.)

#### United Kingdom

Bristol Frenchway Hospital (Molyneux A.J.); Oxford John Radcliffe Hospital (Byrne J., Kerr R.); Plymouth Derriford Hospital (Adams W.); Birmingham University Hospital (Lamin S.); Cardiff University Hospital of Whales (Halpin S.); Edinburgh Royal Infirmary Western General Hospital (White P., Sellar R.); Essex Centre for Neurological Sciences (Chawda S.); Liverpool The Walton Centre (Nahser H., Shaw D.); London Kings College Hospital (Jeffree M.); London University College Hospital (Grieve J., Kitchen N.); Newcastle General Hospital (Gholkar A.); Nottingham Queens Medical Centre (Lenthall R.); Preston Royal Preston Hospital (Patankar T.); Salford Hope Hospital and Manchester Royal Infirmary (Hughes D., Laitt R., Herwadkar A.); Southampton Wessex Neurological Centre (Millar J.); West Sussex Brighton and Sussex University Hospital (Olney J.)

#### Canada

Montréal CHUM Hôpital Notre-Dame (Raymond J., Roy D., Guilbert F., Weill A.); Montreal Neurological Institute (Tampieri D., Mohr G.); Québec Hôpital Enfant-Jésus (Milot G., Gariépy J.L.); Vancouver General Hospital (Redekop G.); Ottawa Hospital (Lum C.); Winnipeg Health Sciences Center (Silvaggio J., Iancu D.); Toronto St Michael's Hospital (Marotta T., Montanera W.)

#### United States

Chicago Rush University Medical Center (Chen M., Lee V., Temes R.); Iowa University of Iowa Hospitals and Clinic (Chaloupka J., Hayakawa M.); Houston The Methodist Hospital (Klucznik RP.); Boston Medical Center - Boston University School of Medicine (Kase C., Lau H.); New York INN Beth Israel (Berenstein A., Niimi Y.); Cornell Medical Centre (Gobin P.); SUNY Downstate Medical Center (Mangla S.); Phoenix Barrow Neurological Institute (McDougall C.); Charleston Medical University of South Carolina (Turk A.); Minneapolis University of Minnesota Medical Center (Tummala R., Qureshi A.)

#### Germany

Dresden Universitatsklinikum Carl Gustav Carus (Von Kummer R.); Hamburg Universitatsklinikum Hamburg-Eppendorf (Zeumer J., Fiehler H.)

#### Italy

Milano Ospedale Niguarda (Valvassori L., Boccardi E., Quillici L.)

#### Norway

Oslo Rikshopitalet University Hospital (Bakke S.J; Kindergaard K.F.)

#### Poland

Warsaw Instytute of Psychiatry and Neurology I Klinika Neurologiczna (Kobayashi A.)

#### Spain

Barcelone Hospital Bellvitge (de Miquel M.A.)

#### Brazil

Rio Grande do Sul Hospital de Clinicas de Porto Alegre (Stefani M.)

#### Hungary

Budapest National Institute of Neurosurgery (Szikora I.; Kulcsar Z.)

## Competing interests

The authors declare that they have no competing interests.

## Authors' contributions

JR and AJM conceived and designed the TEAM study and obtained funding. JR, AJM, TD drafted the manuscript. All authors read and approved the final manuscript.
